# Implementation of change in health care in Sweden: a qualitative study of professionals’ change responses

**DOI:** 10.1186/s13012-019-0902-6

**Published:** 2019-05-14

**Authors:** Per Nilsen, Kristina Schildmeijer, Carin Ericsson, Ida Seing, Sarah Birken

**Affiliations:** 1 Department of Medical and Health Sciences, Division of Community Medicine, Linköping University, SE-581 83 Linköping, Sweden; 2 Department of Health and Care Sciences, Linnaeus University, SE-392 81 Kalmar, Sweden; 3 Cardiology and Speciality Medicine Centre, County Council of Östergötland, SE-581 85 Linköping, Sweden; 4 Department of Behavioural Sciences and Learning, Linköping University, SE-581 83 Linköping, Sweden; 5 Department of Health Policy and Management, Gillings School of Global Public Health, The University of North Carolina at Chapel Hill, 1106F McGavran-Greenberg, 135 Dauer Drive, Campus Box 7411, Chapel Hill, NC 27599-7411 USA

**Keywords:** Change response, Attitude, Health care, Implementation, Evidence-based practice

## Abstract

**Background:**

Implementation of evidence-based practices in health care implies change. Understanding health care professionals’ change responses may be critical for facilitating implementation to achieve an evidence-based practice in the rapidly changing health care environment. The aim of this study was to investigate health care professionals’ responses to organizational and workplace changes that have affected their work.

**Methods:**

We conducted interviews with 30 health care professionals (physicians, registered nurses and assistant nurses) employed in the Swedish health care system. An inductive approach was applied, using a semi-structured interview guide developed by the authors. We used an analytical framework first published in 1999 to analyze the informants’ change responses in which change responses are perceived as a continuum ranging from a strong acceptance of change to strong resistance to change, describing seven forms of change responses along this continuum. Change response is conceptualized as a tridimensional attitude composed of three components: cognitive, affective and intentional/behavioral.

**Results:**

Analysis of the data yielded 10 types of change responses, which could be mapped onto 5 of the 7 change response categories in the framework. Participants did not report change responses that corresponded with the two most extreme forms of responses in the framework, i.e., commitment and aggressive resistance. Most of the change responses were classified as either indifference or passive resistance to changes. Involvement in or support for changes occurred when the health care professionals initiated the changes themselves or when the changes featured their active input and when changes were seen as well founded and well communicated. We did not identify any change responses that could not be fitted into the framework.

**Conclusions:**

We found the framework to be useful for a nuanced understanding of how people respond to changes. This knowledge of change responses is useful for the management of changes and for efforts to achieve more successful implementation of evidence-based practices in health care.

## Introduction

Change is pervasive in health care. Changing disease patterns, aging populations, new discoveries of treatment of diseases, technological advancements, political reforms, and policy initiatives place demands on health care organizations and professionals’ capacity to implement change [1–4]. Health care is facing a wave of new technologies and business models (e.g., telemedicine), which is likely essential if health care is to meet growing needs in areas such as chronic disease management. The patient experience is evolving into a broader patient experience as more patients are becoming involved in decisions about their care, pointing to patient/provider relationships based on partnership and mutual empowerment [5].

Changes within an organization are usually associated with employee psychological uncertainty about how the change will affect their lives [6, 7]. The demand to change has well-documented effects on employee health and well-being, as assessed by a range of indicators and constructs, e.g., work-related stress, mental health problems, change fatigue, poor self-rated health, adverse sleep patterns, sickness absence, hospital admissions, and stress-related medicine prescriptions [6, 8–12].

Health care professionals’ change responses may have important implications for the implementation of evidence-based practices in health care. Theories, models, and frameworks used in implementation science, as well as extant evidence, typically view health care professionals’ responses to the change associated with the implementation of a specific practice, e.g., a particular intervention, program or service. For example, the Consolidated Framework for Implementation Research [13] conceptualizes health care professionals’ change responses as their knowledge and beliefs about the implemented practice. Klein and Sorra [14] describe health care professionals’ responses to changes as the fit between evidence-based practice and health care professionals’ values (“innovation-values fit”). However, changes associated with implementing evidence-based practices represent only one type of change in health care due to the fast pace of change in this sector. Therefore, more general change responses, e.g., negative experiences from numerous and/or large organizational changes impacting health care professionals’ work, may be an important underlying influence on health care practitioners’ implementation intentions and behaviors.

Change responses have usually been described in terms of resistance to change, a concept which was introduced by Coch and French [15]. However, later conceptualizations have expanded beyond conceptualizations of resistance to change to encompass various degrees of acceptance (or readiness) for change, thus creating a continuum of change responses. Coetsee [16] describes seven forms of change responses, from aggressive resistance, active resistance and passive resistance to indifference, support, involvement, and commitment (please see further details in Table 1). Change response in the framework is conceptualized as a tridimensional attitude composed of three components: cognitive (opinions about changes, their usefulness, advantages and disadvantages, etc.), affective (feelings about changes), and intentional/behavioral (actions already taken or which will be taken for or against changes). This tridimensional concept of change response was proposed by Elizur and Guttman [17] and is widely used [18].

**Table 1:** Coetsee’s framework of change responses [16]

Forms of response	Description
Commitment	Powerful acceptance of change, requiring employee empowerment, and a recognition of their contributions and efforts, as well as employees’ understanding and acceptance of values and goals for achieving the organization’s mission
Involvement	Strong form of acceptance of changes, which involves taking part in or “doing” the changes, as manifested by willing cooperation and participative behaviors
Support	Expressing a positive view of changes but it does not mean that one acts to promote or participate in the changes
Indifference	A neutral or transition zone that lies between the three forms of acceptance (commitment, involvement, and support) and three forms of resistance (passive, active, and aggressive resistance). It is also described as a (fourth) form of resistance to change, but it is characterized by fairly neutral attitudes and passive resignation. It involves a lack of positive or negative attitudes and no behavioral responses to changes
Passive resistance	Mild opposition to changes, demonstrated by voicing negative views and considering quitting the job
Active resistance	Strong opposition to changes, displayed through negative attitudes and impeding behaviors such as protesting
Aggressive resistance	Destructive opposition to change, which may involve active efforts to hinder change, such spreading of destructive rumors, strikes, subversion, and sabotage

Understanding health care professionals’ change responses may be critical for facilitating implementation to achieve an evidence-based practice in the rapidly changing health care environment. However, responses to change in health care remain circumscribed in implementation theory, as described above, and poorly understood empirically. Hence, the aim of this study was to investigate health care professionals’ responses to organizational and workplace changes that have affected their work. We used Coetsee’s analytical framework to categorize different change responses. Knowledge of health care professionals’ change responses could be important to identify opportunities for promoting acceptance to change and limiting resistance to change inherent in the implementation of evidence-based practices in health care.

## Methods

### Study setting, design, and participants

We conducted interviews with 30 health care professionals (physicians, registered nurses, assistant nurses) employed in the Swedish health care system, which is mainly publicly funded although private health care also exists. All residents are insured by the state, with equal access for the entire population. Out-of-pocket fees are low and regulated by law.

We used a purposeful sampling strategy to achieve a heterogeneous sample of health care professionals working in different health care facilities in Sweden, ranging from primary care to hospital and tertiary care, with patients who varied in terms of health status and duration of stay. The aim was to achieve a sample of health care professionals that represented a broad spectrum of perceptions and experiences concerning changes in health care.

We recruited health care professionals through an e-mail that briefly described the study. The e-mail request was sent to the manager of each work unit, with a request that they forward our request to physicians, registered nurses and assistant nurses. To those who responded, we then sent an informational letter describing the study. None declined involvement after receiving the information letter. We scheduled interviews at a time (between January and September 2018) and in a location of participants’ choosing where they could feel comfortable speaking honestly (e.g., office with a closed door).

### Data collection

The data collection applied an inductive approach, using a semi-structured interview guide developed by the authors. We generated the questions based on the existing literature on organizational change and change reactions [16, 18, 19]. The questions concerned the participants’ experiences and perceptions of any changes that they considered to have affected their work, regardless of whether these changes were “objectively” large, e.g., a restructuring of the organization, or small, e.g., modification of an already existing documentation routine, covering both broader, more general changes and more specific examples of changes, e.g., merging of the informant’s work unit with another unit, changed work routines or tasks, introduction of new information technology systems and moving to new localities. We did *not* ask about specific changes or provide examples, but instead allowed the participants to discuss any organizational and workplace changes they considered to have relevance for their work. This conceptualization of change is based on the fact that every individual experience change in a unique way; the same change may be attractive and imply advantages for some and be a source of stress and disadvantages for others [18].

We pilot-tested the questions in two interviews with regard to meaningfulness for participants and clarity of concepts. The pilot interviews indicated that the questions were generic enough to be used in different health care contexts, that the wording was clear, and that the interview did not exceed 60 min (which was deemed to be a maximum considering the participants’ work schedule). Individual interviews were conducted by all the authors except for SB, who does not speak Swedish, and were digitally recorded. Each interview lasted between 28 and 104 min, with an average of approximately 50 min. The interviews were transcribed verbatim by a professional transcription agency and were then inspected by the researcher who conducted the interview.

Before starting an interview the participant was asked to re-read the information letter and give a written informed consent to participate. Each interview began with some questions about the participant, the content of his or her work and some information about the workplace. This was followed by questions about what changes have taken place in the workplace, including the participant’s experiences and perceptions concerning the extent and nature of the changes and whether there was an increase or decrease over time. The participant was asked to provide examples of both successful and unsuccessful changes, from their perspective. We then asked questions about the participant’s response to the different changes. The interview guide ended with questions on potential and actual strategies to reduce negative health and well-being consequences of change and a final question concerning whether the participant had anything to add to what had been discussed.

### Data analysis

We used the analytical framework by Coetsee [16] to analyze health care professionals’ change responses. Coetsee conceives change responses as a continuum ranging from a strong acceptance of the change to strong resistance to change. He describes seven forms of change responses along this continuum (Table 1).

To compare and contrast our study findings to the Coetsee framework, we began with qualitative content analysis, a technique for analysis of texts grounded in empirical data with an explorative and descriptive character [20]. As a first step, PN, CE, and KS read all transcripts to obtain an understanding of the whole. PN, CE, and KS then individually coded the transcripts using content analysis, which entails a structured analysis process to code and categorize the data. Next, we highlighted words in the text that captured various key statements and thoughts in relation to the study aim. We then aggregated the codes into clusters based on the similarity of the content and their relation to each other [21].

After re-examination in two group meetings, PN, CE, and KS merged the initial clusters into categories and labeled them [21]. We then cross-examined the categories to ascertain that they were defined in such a way that they were internally as homogeneous as possible and externally as heterogeneous as possible [20]. We then independently analyzed the data and compared our findings. Next, we mapped the categories onto the different types of response specified in the Coetsee framework, discussing our findings until we achieved no inconsistencies and a shared understanding [22].

PN, CE, and KS identified representative quotations for reporting. PN, who is fluent in English, then translated quotations from Swedish to English. PN, CE, and KS examined the quotations for accuracy. Finally, SB, whose first language is English, reviewed the English-language quotations for clarity.

## Results

We conducted semi-structured individual interviews with 30 health care professionals: 11 physicians, 12 registered nurses and seven assistant nurses (Table 2). The participants were employed in 6 different health care units, all located in cities, with 67,000, 135,000, and 150,000 inhabitants, respectively, all located in the south-east part of Sweden.

**Table 2:** Participant characteristics

Characteristics	Physicians (*N* = 11)	Registered nurses (*N* = 12)	Assistant nurses (*N* = 7)
Sex, *n* (%)			
Male	5 (45.5)	1 (8.3)	1 (14.3)
Female	6 (54.5)	11 (91.7)	6 (85.7)
Years of practice, *n* (%)			
0–9 years	0 (0)	1 (8.3)	0 (0)
10–20 years	7 (63.6)	3 (25)	0 (0)
21 years or more	4 (36.4)	8 (66.7)	7 (100)
Median years of practice	17 years	28 years	30 years
Years in the health care facility, *n* (%)			
0–9 years	4 (36.4)	6 (50)	4 (57.1)
10–20 years	5 (45.4)	3 (25)	3 (42.9)
21 years or more	2 (18.2)	3 (25)	0 (0)
Median years in the health care facility	17 years	9 years	9 years
Health care facility, *n* (%)			
Medical unit	3 (27.3)	4 (33.3)	2 (28.6)
Surgical unit	1 (9.0)	1 (8.3)	1 (14.3)
Orthopedic unit	2 (18.2)	1 (8.3)	1 (14.3)
Emergency care unit	2 (18.2)	1 (8.3)	1 (14.3)
Primary health care center	3 (27.3)	3 (25.0)	2 (28.6)
Hospital management	0 (0)	2 (16.7)	0 (0)

Analysis of the data yielded 10 types of change responses, numbered 1 to 10 below and in Table 3. The different types of responses could be mapped onto five of the seven change response categories in Coetsee’s framework [16]. Participants did not report change responses that corresponded with Coetsee’s two most extreme forms of responses, i.e., commitment and aggressive resistance. Several representative quotes for each type of change response are provided in Table 3.

**Table 3:** Participants’ change responses

Forms of response (Coetsee)	Categories identified in the data	Quotes from the data (P, physician; RN, registered nurse; NA, assistant nurse)
Involvement	1. Engaging in bottom-up changes	“When changes are gradual and initiated by ourselves, I never see them as a problem” [P13]
		“I’m very positive to changes, but then changes have to emerge from the bottom up. ... We launched a nurse unit and it was really good. We sort of had the chance to develop and learn ourselves.” [RN5]
		“When decisions are taken in the management group which are not anchored in the work units, such changes are not good. They generate frustration.” [RN12]
		“The biggest changes always come from above, and they are always the ones that are most problematic.” [NA15]
Support	2. Supporting well-founded changes	“Changes where you immediately think, ‘This is not grounded in reality’, create a lot of negative emotions. ... Most people handle changes well, they accept changes, if you only understand why [they are made]. [It is important] to explain so you have an understanding of why an initiative is important.” [P9]
		“The condition to go along with a change is that you feel it has some value.” [P13]
		“If you do not understand the value, you do not understand the purpose, and then you think, ‘Why are they making these changes? Why is this a priority now?’ And you often know that it is only temporary, then they change and put the money into something else.” [RN30]
		“A change where you cannot influence what happens is really bad, of course, and that you do not get an explanation for why it happens, or that you do not understand why it happens. It generates so much bitterness, so much resentment and many become dejected.” [NA11]
	3. Supporting well-communicated changes	“All must feel that they have received information and that they have been allowed to express an opinion, to be able to influence [the changes].” [RN25]
		“Some changes are tough, when you are not prepared. I mean, computer systems and big things like that, where you are prepared, they have put in an effort before [the change]. But these other changes, we changed telephone systems, you do not get any information, you are in the dark.” [RN30]
		“It’s all about information, to know why we should change, this new apparatus should be introduced or this system, etc. [It’s important] to get sufficient information that this [change] will lead to this improvement or that patients’ situation is much improved or you get improvements.” [NA 15]
Indifference	4. Experiencing change apathy	“In one way, I get less agitated today than I did 10 years ago. Because then I was massively disappointed for everything that did not turn out well. Today I’m a bit resigned.” [P9]
		“Resistance is not to bother with this [i.e. the changes], but maybe that’s not resistance. That’s probably more passivity.” [P13]
		”Many are a bit resigned and tired and think, ‘Yes, I wonder what will become of this [change] this time, then?’” [RN5]
		“Well, you let go of all engagement, you let go of all reflection, you let go really of everything that has not to do with my own person. And at the same time you build a shell around yourself.” [NA11]
	5. Experiencing physical responses to changes	“I think this ‘change fatigue syndrome’ has spread like wildfire. It concerns people who consciously or unconsciously are not working in accordance with their values.” [P9]
		“I used to think, ‘Oh, great fun!’ But that’s not the way any longer; instead I feel like this, ‘Oh, well, how tiresome! Tough!’ I feel that I become disengaged. … So you get disappointed and think that ‘No, damn, I do not give a shit. I’m not going to become engaged. I do not have the stamina to do it one more time.’” [RN5]
		”You often share this tiredness. If there are many in a group who are tired of changes, who do not have the energy to think positively about things, then you share it in the workgroup and it can turn into endless whining at the coffee table.” [RN16]
	6. Experiencing emotional responses to changes	“There are many who have hit the wall, some sort of reaction, post-traumatic stress. The feelings are very strong, very negative and a lot of stress [is involved]. ... A lot of strong feelings and bitterness and anxiety, stress, anger, frustration.” (P3)
		“These [i.e., changes] create stress, right? There are changes, they change something and then you have hardly had the time to accomplish change, [for example] to start a new way of working, before they change again.” [P17]
		“The joy of working disappears. It’s not fun to go to work.” [RN21]
		“It [i.e. the change] created anxiety and a sense of insecurity among the employees.” [RN25]
Passive resistance	7. Complaining about changes	“The digitalization of the medical records, as a whole, was a process that caused many to curse, sigh, and believe was so bad.” [P6]
		“A lot of negativity is accumulated and, like, too much whining.” [P24]
		“You go there and complain and that’s not so good since you contaminate other people with it.” [P29]
		“It turned into a lot of fuss and speculation, but it affected everyone.” [NA26]
	8. Reducing work effort in response to changes	“It’s even so that you go backwards and try to do as little as possible and only what is absolutely necessary that you perceive that you must do in your job.” [P8]
		“It’s like this, ‘Now we should do this.’ OK, I’ll do it, but with the least amount of effort.” [RN5]
		”Why do I have to make an effort? It’s better that I work slower.” [RN25]
		”You have to use different strategies to handle it, everything from completely disconnecting to doing what you have to do, but not more, eight hours a day.” [NA11]
	9. Considering quitting the job in response to changes	“And then you start thinking, like, ‘What the heck are we doing?’ That’s when you start thinking about retirement or doing something else.” [P23]
		“No, I do not have the energy any more, I quit.” [RN4]
		“Then I was so tired and felt I did not get any recognition from bosses for working so hard and, well, I did not feel so very good. Sad but that’s a part of the reason why I changed jobs.” [RN10]
		“Of course, you would have distanced yourself from these circumstances if you had been able. That’s the way it was, but the labour market wasn’t like that.” [NA11]
Active resistance	10. Avoiding involvement in changes	“I do not get angry and goes around screaming and shouting, but I do get agitated. Until I get dejected. And then I try to avoid having anything to do with that change.” [P8]
		“That’s what we have to relate to. There are no alternatives, but sometimes I do not give a damn, because I do not have the time, I cannot stand it today, to do that. I do not give a hoot.” [P29]
		“I do not take it in, I cannot stand it. And then you get all these mails with information that, ‘Now we will do this or that. Now this will change and here is the starting date for that.’ So I just get involved in what concerns me.” [RN5]

### Involvement in changes

(1) Involvement was the strongest form of support for changes expressed by the health care professionals. They provided numerous statements that suggested engagement in changes that were initiated by the health care professionals themselves or were characterized by their active input. They said that engagement was premised on changes that were “initiated by [themselves],” which would “emerge from the bottom up.” This engagement was not expressed in relation to top-down changes initiated by managers or politicians that lacked their own input. Rather, these changes generated “frustration” and were considered the “most problematic.”

### Support for changes

(2) The health care professionals also expressed support for changes that they viewed as well founded because they could see the necessity or utility of the changes. The importance of having “an understanding” of why a change was made or prioritized was emphasized. In contrast, changes that were viewed as more or less pointless, e.g., not being “grounded in reality,” or appearing to be a waste of time and resources did not garner their support.

(3) Support was also expressed by the health care professionals for changes that they considered well communicated and predictable, which allowed them to prepare. This was summarized by one participant as “it’s all about information.” However, this support was not conveyed for changes that they did not receive sufficient information about. For example, one participant described being “in the dark” about a change. Similarly, health care professionals did not support changes that were perceived to be abrupt or unexpected.

### Indifference to changes

(4) The health care professionals communicated change apathy, i.e., a state of indifference, when they talked about responses to many changes. This apathy manifested itself in numerous ways. Some health care professionals voiced a lack of interest, disengagement, or resignation in response to changes. They mentioned their “passivity” as well as being “resigned and tired” and not trying to “bother” with the changes.

(5) Many statements by the health care professionals indicated physical responses to changes such as exhaustion and weariness. Some talked about “tiredness” and not having “the stamina” to endure more changes. One participant even talked about suffering from “change fatigue syndrome.”

(6) Indifference also revealed itself in health care professionals’ emotional reactions to changes. They described negative emotions, including “very strong, very negative” feelings, “anxiety and a sense of insecurity.” These emotions generated anger and frustration, inducing stress. There was even a mention of “post-traumatic stress” as an emotional change response.

### Passive resistance to changes

(7) Some statements by the health care professionals suggested passive resistance to changes. This revealed itself in voicing complaints and expressing dissatisfaction with changes by means of “too much whining.” The changes caused “many to curse, sigh” and they created “a lot of fuss and speculation.” These reactions seemed to spread and could “contaminate other people” in the work environment.

(8) Another expression of passive resistance among some health care professionals was to respond to changes by consciously reducing one’s own work efforts and ambitions. Participants mentioned that they would “try to do as little as possible” and work with “the least amount of effort” in response to changes.

(9) A few health care professionals also expressed passive resistance to changes by considering quitting their job in health care altogether, e.g., “thinking about retirement or doing something else.”

### Active resistance to changes

(10) Some health care professionals responded to changes with active resistance by actively removing themselves from having to be involved in the changes in question. This was expressed by the participants in terms of trying “to avoid having anything to do” with the changes and refusing to “take [the changes] in.”

## Discussion

Implementation of evidence-based practices in health care implies change [23]. In practice, health care professionals typically face many concurrent changes [24], and individuals’ responses to these changes vary [18].

We found Coetsee’s [16] change responses framework to be useful for a nuanced understanding of how people respond to changes, describing responses that range from a strong acceptance of the change to strong resistance to change. We identified in our study 10 types (i.e., sub-categories) of change responses, which could be mapped onto five of the seven response categories of Coetsee’s framework. We did not identify any change responses that could not be fit into the framework.

The participants did not report change responses that corresponded with Coetsee’s two most extreme forms of responses, i.e., *Aggressive resistance* and *Commitment.* The lack of change responses characterized by *Aggressive resistance* may be attributed to the Swedish workplace culture, including health care, which typically promotes stability and favors consensus over arguing or expressing strong emotions or opinions [25]. *Commitment* is a powerful acceptance of change which has been described by the willingness of employees to direct their energy and loyalty to the benefit of the organization to such an extent that a strong attachment is created to the values, goals, and vision of the organization [26, 27]. The lack of statements that conveyed this type of change response can be explained with reference to the overall paucity of examples of change responses categorized as *involvement* or *support*, i.e., the two less enthusiastic forms of change acceptance. These findings suggest that health care professionals are insufficiently engaged in efforts to solicit their commitment, which means that it may be unrealistic to expect a strong commitment from health care professionals when implementing change, including evidence-based practices.

The two forms of change acceptance we identified, involvement and support, were generally associated with changes that were initiated by the health care professionals themselves or featured their active input, changes they viewed as well-founded because they could see the utility of the changes or changes they considered well communicated and predictable. These findings are in line with previous organizational research which shows that resistance to change is more likely if employees consider a change initiative pointless and do not have a say in the planning or implementation of the change, while acceptance of change is more likely if they consider the change to be sensible and respect the individuals behind the change initiative [19]. How change is carried out is important, with open strains of communication and leadership that is perceived as competent and truthful in its implementation of change increasing the chances of change acceptance [18]. In line with this, research has demonstrated that organizational changes cause stress when changes create uncertainty (e.g., [28, 29]), are poorly communicated (e.g., [30]), are considered unfair (e.g., [31, 32]), and take place too quickly or too slowly (e.g., [32]).

Organizational theorists have acknowledged that sense-making processes are essential to understanding individuals’ responses to change [24, 33]. Interestingly, the characteristics of changes associated with involvement and support are consistent with Antonovsky’s sense of coherence theory [34], which can be applied at different system levels, from the individual to the societal level. The theory posits that we constantly are exposed to changes that function as stressors. A sense of coherence, therefore, reflects a coping capacity to deal with stressors and consists of comprehensibility, manageability, and meaningfulness. Changes which are seen as well founded, well communicated, and predictable are likely viewed as comprehensible, changes that are initiated by health care professionals or involve their active input are perceived as manageable, and changes seen as well founded are considered meaningful. The sense of coherence concept has been applied in many studies, e.g., concerning stress, burnout, and working circumstances [35], but to our knowledge the three elements of comprehensibility, manageability, and meaningfulness have not been applied in organizational research to provide understanding of why certain changes might be more successful than others. Further research is warranted to explore the extent to which implementation of evidence-based practices are perceived as comprehensible, manageable and meaningful. Future research should also assess the influence of variables such as involvement in planning, quality of communication regarding changes, and perceived relevance of changes on change responses. Research is also needed to find out whether these conditions have an additive or interactive effect on commitment.

Three of the 10 types of change responses were mapped onto the *indifference* category. This is considered a zone between acceptance and rejection of change in Coetsee’s framework, characterized by neutral cognitive and affective responses and passively resigned behaviors. Change apathy seemed to be a particularly common response to changes among health care professionals who had previously experienced changes impacting on their work which they considered unsuccessful. The organizational literature usually purports, with some empirical support, that 70% of all organizational change initiatives are failures [36]. Perceiving many organizational and workplace change initiatives as unsuccessful can yield change cynicism [37], which represents feelings that often combine pessimism about the likelihood of successful change with the blame of those responsible for change as incompetent [38]. Change cynicism appears to be a reaction to experiences from within an organization rather than being a general characteristic or trait [7]. Health care professionals who experience change cynicism are unlikely to have positive responses to changes involving the implementation of evidence-based practices.

Another sub-category of indifference, physical responses were described in terms of tiredness or change fatigue, which is exhaustion associated with feelings of being drained and depleted beyond one’s capacity to handle workplace demands and everyday work tasks [39, 40]. Change fatigue is different from various forms of change resistance since the behaviors are often passive, whereas change resistance behaviors are intentional. With change fatigue, individuals become disengaged and do not express their dissent about changes. Because of this passive behavior, change fatigue often is undetected by managers and leaders in organizations [41]. Research suggests that new graduate health care professionals and professionals newly transferred to a unit are more vulnerable to change fatigue [42]. Implementation of evidence-based practices in settings where change fatigue is prevalent can be expected to be difficult.

Physical responses seemed to be intertwined with emotional responses, with the health care professionals reporting a range of emotions, from anxiety and stress to frustration and anger. It is noteworthy that emotional change responses are not highlighted in descriptions of the Coetsee framework [16], but they clearly played an important role among the participants of this study. Emotional responses were only expressed in relation to change resistance and not with regard to change acceptance, i.e., involvement or support. It has been argued that affective aspects of change responses have been overlooked, although both theoretical and empirical studies point to the relevance of the affective element of attitudes [11]. This also has relevance for the implementation of evidence-based practices. For example, a Swedish study found that it was challenging to implement evidence-based palliative care in nursing homes as the desired behavior, providing existential care for the dying, was emotionally charged and presented difficulties even after the staff’s participation in educational interventions to acquire necessary skills and knowledge [43].

We also identified statements attributable to *passive resistance*. Resistance is the most studied response to change [44], being viewed as any set of intentions and actions that slows down or hinders the implementation of change [45]. Passive resistance revealed itself in reduced work effort, something which could potentially limit the effectiveness and efficiency of implemented evidence-based practices because policies and procedures may not be followed when delivering them, thus limiting their potential benefit. Implementation failure may result in type III error, i.e., attributing null results to an evidence-based practice’s inherent lack of effectiveness when, in actuality, the null results are due to implementation failure [46].

Passive resistance was also expressed in terms of health care professionals’ complaints about changes and thoughts about quitting the job in response to changes. Discontent was expressed by all three professional groups of the study, but physicians more often than registered nurses and assistant nurses complained about their working conditions. Although this response was passive, the participants described how negativity could spread and affect others, thus likely contributing to a culture of discontent that can have negative effects on the productivity.

*Active resistance* was expressed by some health care professionals who stayed away from changes or limited their involvement by trying to ignore the changes they did not want to be affected by. This “avoidance” strategy also seemed to be more common among the physicians than among the other professions.

Our findings concerning passive resistance and active resistance are aligned with research that has shown that physicians often are dissatisfied with their job, which can have negative consequences for their productivity, intent to leave the job, work ability, and amount of sick leave days [47–49]. The increased workload in combination with reduced autonomy has been identified as key sources of this dissatisfaction [50]. Physicians tend to be critical toward managerial control of their work [51–53] and are often reluctant to become involved in management-initiated quality improvement initiatives [4]. The central role of physicians for implementation of evidence-based practices in health care is well recognized, as they often act as informal leaders in daily health care practice, functioning both as change agents and gatekeepers to desired changes [54, 55].

Some methodological issues must be considered when interpreting the findings. A qualitative approach was chosen because little is known about change responses in Swedish health care. Interviews with physicians, registered nurses, and assistant nurses were considered the most relevant method for collecting information and gaining a deeper understanding of the topic. The change responses identified in this study are not intended as an exhaustive list of all possible responses; other studies may yield different responses or give different priorities to other factors. The results cannot be directly transferred to other health care settings in Sweden or internationally.

We used Coetsee’s [16] framework to analyze different change responses. According to De Casterle et al. [56], using a preconceived framework runs the risk of prematurely excluding alternative ways of organizing the data that may be more illuminating. However, we did not use Coetsee’s framework to inform the questions presented to the participants, and it was not applied until the second phase of the data analysis, after the data had first been analyzed inductively to arrive at change responses. Choosing one theory, model or framework often means placing weight on some aspects at the expense of others, thus offering only partial understanding [57]. However, Coetsee’s framework [16] was found to be sufficiently broad to allow for a fairly inductive approach. Some of the change response categories were difficult to distinguish from each other, including active and passive change responses although the former category involved taking some sort of action in response to changes whereas the latter category did not. We have sought other studies that may have applied Coetsee’s framework [16] to empirical studies, but we have not been able to find any. Given the usefulness of Coetsee’s framework for understanding change responses, we recommend that the framework be used in future implementation research on change responses.

The multidisciplinary research team was a strength of the study, because it permitted different perspectives on the issue of change responses in health care. The team consisted of the following professions: registered nurse (KS), behavioral scientist (CE), political scientist (IS), behavioral economist (PN), and organizational sociologist (SB). Another strength was the relatively high number of interviews. This allowed us to use quotations from many different participants, which added transparency and trustworthiness to the findings.

In terms of implications for implementation science, our study suggests that change responses may be an underlying explanation for some of the barriers for successful implementation of evidence-based practices often described in implementation research, e.g., lack of awareness, insufficient motivation, negative attitudes or ingrained habits among health care professionals [54]. Change responses may be associated with and/or influence implementation constructs such as receptive context for change [58], readiness for change [13] and tension for change [13, 58, 59], and optimism and beliefs about consequences and capabilities, as described in Theoretical Domains Framework [60]. Also, health care professionals’ change responses may be analogous to change commitment in the Organizational Readiness to Change theory [23]. Coetsee’s framework offers a nuanced way of understanding changes involved in implementation of evidence-based practices, but further research is needed to explore the relationship between change responses and various implementation constructs.

The study also points to the importance of the “timing” of implementing evidence-based practices, i.e., when implementation occurs. While lack of time is described as a barrier in many implementation studies and determinant frameworks (e.g., [61–63]), the temporality of implementation in relation to other changes seems neglected despite the fact that health care professionals usually face many concurrent changes of relevance for their work. Further, implementation studies typically investigate one change, i.e., one evidence-based practice, at a time rather than viewing changes, including those required when implementing evidence-based practices, in a broader perspective of many simultaneous changes.

## Conclusion

In conclusion, this study of health care professionals’ change responses identified 10 types of change responses, which could be mapped onto five of the seven response categories of a framework developed by Coetsee [16]. We found that the many changes are met with indifference or passive resistance. Changes are more likely to be accepted if they are initiated by the health care professionals themselves or feature their active input and when changes are well founded and well communicated. This is valuable knowledge for use in the management of changes and for efforts to achieve more successful implementation of evidence-based practices in health care.

## References

[1] Kuhlmann E (2006). Modernising healthcare.

[2] Pollitt C, Bouckaert G (2004). Public management reform: a comparative analysis.

[3] Gray M. Evidence-based healthcare and public health. Edinburgh: Churchill Livingstone; 2009.

[4] Gadolin C. The logics of healthcare – in quality improvement work. Gothenburg: University of Gothenburg; 2017.

[5] Thimbleby H (2013). Technology and the future of healthcare. J Public Health Res.

[6] Rafferty A (2006). Griffin M (2006). Perceptions of organizational change: a stress and coping perspective. J Appl Psychol.

[7] Grama B, Todericiu R (2016). Change, resistance to change and organizational cynicism. Stud Bus Econ.

[8] Kuokkanen L, Suominen T, Härkönen E, Kukkurainen ML, Doran D (2009). Effects of organizational change on work-related empowerment, employee satisfaction, and motivation. Nurs Admin Q.

[9] Bamberger GS, Vinding AL, Larsen A, Nielsen P, Fonager K, Nielsen RN (2012). Impact of organisational change on mental health: a systematic review. Occup Environ Med.

[10] Benach J, Vives A, Amable M, Vanroelen C, Tarafa G, Muntaner C (2014). Precarious employment: understanding an emerging social determinant of health. Annu Rev Public Health.

[11] Rafferty AE (2016). Jimmieson NL subjective perceptions of organizational change and employee resistance to change. Br J Manag.

[12] Yu M (2009). Employees’ perception of organizational change. Public Pers Manag.

[13] Damschroder LJ, Aron DC, Keith RE, Kirsh SR, Alexander JA, Lowery JC (2009). Fostering implementation of health services research findings into practice: a consolidated framework for advancing implementation science. Implement Sci.

[14] Klein KJ, Sorra JS (1996). The challenge of innovation implementation. Acad Manag Rev.

[15] Coch L, French JRP (1948). Overcoming resistance to change. Hum Relat.

[16] Coetsee L (1999). From resistance to commitment. Public Adm Q.

[17] Elizur D, Guttman L (1976). The structure of attitudes toward work and technological change within an organization. Adm Sci Q.

[18] Oreg S, Vakola M, Armenakis A (2011). Change recipients’ reactions to organizational change. J Appl Behav Sci.

[19] Hill LA (2009). Managing change.

[20] Krippendorf K (2013). Content analysis. An introduction to its methodology.

[21] Hsieh HF, Shannon SE (2005). Three approaches to qualitative content analysis. Qual Health Res.

[22] Patton MC (2015). Qualitative research and evaluation methods. Thousand oaks: sage.

[23] Weiner BJ (2009). A theory of organizational readiness for change. Implement Sci.

[24] Loretto W, Platt S, Popham F (2010). Workplace change and employee mental health: results from a longitudinal study. Br J Manag.

[25] Alharbi TSJ, Ekman I, Olsson L-E, Dudas K, Carlström E (2012). Organizational culture and the implementation of person centered care: results from a change process in Swedish hospital care. Health Policy.

[26] Allen NJ, Meyer JP (1990). The measurement and antecedents of affective continuance and normative commitment in the organization. J Occup Psychol.

[27] Jaros SJ, Kochler JM, Sincich T, Wall JL, Jauch JR (1991). Effects of calculative affective and moral commitment on the turnover process: evaluation of three structural equation models. Best paper proceedings, 51st academy of management meeting, Miami Beach, august.

[28] Paulsen N, Callan VJ, Grice TA, Rooney D, Gallois C, Jones E (2005). Job uncertainty and personal control during downsizing: a comparison of survivors and victims. Hum Relat.

[29] Lawrence SA, Callan VJ (2010). The role of social support in coping during the anticipatory stage of organizational change: a test of an integrative model. Br J Manag.

[30] Riolli L, Savicki V (2006). Impact of fairness, leadership, and coping on strain, burnout and turnover in organizational change. Int J Stress Manag.

[31] Fugate M, Prussia GE, Kinicki AJ (2012). Managing employee withdrawal during organizational change: the role of threat appraisal. J Manag.

[32] Smollan RK, Ashkanasy NM, Hartel CEJ, Zerbe WF (2012). Emotional responses to the injustice of organizational change: a qualitative study. Research on emotion in organizations.

[33] George JM, Jones GR (2001). Towards a process model of individual change in organizations. Hum Relat.

[34] Antonovsky A (1987). Unraveling the mystery of health: how people manage stress and stay well. The Jossey-Bass social and behavioral science series and the Jossey-Bass health series.

[35] Ward M, Schulz M, Bruland D, Lohr M (2014). A systematic review of Antonovsky’s sense of coherence scale and its use in studies among nurses: implications for psychiatric and mental health nursing. J Psychiatr Nurs.

[36] Beer M, Nohria N. Cracking the code of change. Harvard Bus Rev. 2000;May-June:133–41. 11183975

[37] Anderson LM (1996). Employee cynicism: an examination using a contract violation framework. Hum Relat.

[38] Reichers AE, Wanous JP, Austin JT (1997). Understanding and managing cynicism about organisational change. Acad Manag Exec.

[39] Halbesleben JRB, Buckley MR (2004). Burnout in organisational life. J Manag.

[40] Bernerth J, Walker H, Harris S (2011). Change fatigue. Work Stress.

[41] McMillan K, Perron A (2013). Nurses amidst change: the concept of change fatigue offers an alternative perspective on organizational change. Policy Polit Nurs Pract.

[42] Vestal K (2013). Change fatigue: a constant leadership challenge. Nurs Leader.

[43] Nilsen P, Wallerstedt B, Behm L, Ahlström G (2018). Towards evidence-based palliative care in nursing homes in Sweden: a qualitative study informed by the organizational readiness to change theory. Implement Sci.

[44] Brown R, Wey H, Foland K (2018). The relationship among change fatigue, resilience, and job satisfaction of hospital staff nurses. J Nurs Scholarsh.

[45] del Val MP, Fuentes CM (2003). Resistance to change: a literature review and empirical study. Manag Decis.

[46] Mayer DK, Birken SA, Chen RC (2015). Avoiding implementation errors in cancer survivorship care plan effectiveness studies. J Clin Oncol.

[47] Dewa CS, Loong D, Bonato S, Thanh NX, Jacobs P (2014). How does burnout affect physician productivity? A systematic literature review. BMC Health Serv Res.

[48] Linzer M, Levine R, Meltzer D, Poplau S, Warde C, West CP (2014). 10 bold steps to prevent burnout in general internal medicine. J Gen Intern Med.

[49] Schrijver I (2016). Pathology in the medical profession? Taking the pulse of physician wellness and burnout. Arch Pathol Lab Med.

[50] Pedrazza M, Berlanda S, Trifiletti E, Bressan F (2016). Exploring physicians' dissatisfaction and work-related stress: development of the PhyDis scale. Front Psychol.

[51] Landon BE, Aseltine R, Shaul JA, Miller Y, Auerbach BA, Cleary PD (2002). Evolving dissatisfaction among primary care physicians. Am J Manag Care.

[52] Suter WL (2008). The physician’s role in patient safety: what’s in it for me? Proc. Bayl Univ Med Cent.

[53] Eriksson N, Mullen T, Andersson T, Gadolin C, Tengblad S, Ujvari S (2016). Involvement drivers: a study of nurses and physicians in improvement work. Qual Manag Health Care.

[54] Growl R, Wensing M, Eccles M (2005). Improving patient care: the implementation of change in clinical practice.

[55] Berghout MA, Fabbricotti IN, Buljac-Samardzic M, Hilders CGJM (2017). Medical leaders or masters? – a systematic review of medical leadership in hospital settings. PLoS One.

[56] De Casterle DB, Gastmans C, Bryon E, Denier Y (2012). QUAGOL: a guide for qualitative data analysis. Int J Nurs Stud.

[57] Nilsen P (2015). Making sense of implementation theories, models and frameworks. Implement Sci.

[58] Greenhalgh T, Robert G, Bate P, Macfarlane F, Kyriakidou O (2005). Diffusion of innovations in service organisations: a systematic literature review.

[59] Gurses AP, Marsteller JA, Ozok AA, Xiao Y, Owens S, Pronovost PJ (2010). Using an interdisciplinary approach to identify factors that affect cliniciansʼ compliance with evidence-based guidelines. Crit Care Med.

[60] Cane J, O’Connor D, Michie S (2012). Validation of the theoretical domains framework for use in behaviour change and implementation research. Implement Sci.

[61] Cabana MD, Rand CS, Powe NR, Wu AW, Wilson MH, Abboud PAC (1999). Why don't physicians follow clinical practice guidelines? A framework for improvement. JAMA..

[62] Fleuren M, Wiefferink K, Paulussen T (2004). Determinants of innovations within health care organizations. Int J Qual Health Care.

[63] Nutley SM, Walter I, Davies HT (2007). Using evidence: how research can inform public services.

